# Surface Modification of Monolayer MoS_2_ by Baking for Biomedical Applications

**DOI:** 10.3389/fchem.2020.00741

**Published:** 2020-10-06

**Authors:** Yan Wang, Yuanjun Ma, Jinping Shi, Xiangyu Yan, Jun Luo, Huilong Zhu, Kunpeng Jia, Juan Li, Can Yang Zhang

**Affiliations:** ^1^School of Physics, Beijing Institute of Technology, Beijing, China; ^2^School of Optics and Photonics, Beijing Institute of Technology, Beijing, China; ^3^Institute of Microelectronics, Chinese Academy of Sciences, Beijing, China; ^4^Antimicrobial Resistance Interdisciplinary Research Group, Singapore-MIT Alliance for Research and Technology, Singapore, Singapore

**Keywords:** MoS_2_, surface modification, p-type doping, baking, biomedical application

## Abstract

Molybdenum disulfide (MoS_2_), a transition metal dichalcogenide material, possesses great potential in biomedical applications such as chemical/biological sensing, drug/gene delivery, bioimaging, phototherapy, and so on. In particular, monolayer MoS_2_ has more extensive applications because of its superior physical and chemical properties; for example, it has an ultra-high surface area, is easily modified, and has high biodegradability. It is important to prepare advanced monolayer MoS_2_ with enhanced energy exchange efficiency (EEE) for the development of MoS_2_-based nanodevices and therapeutic strategies. In this work, a monolayer MoS_2_ film was first synthesized through a chemical vapor deposition method, and the surface of MoS_2_ was further modified via a baking process to develop p-type doping of monolayer MoS_2_ with high EEE, followed by confirmation by X-ray photoelectron spectroscopy and Raman spectroscopy analysis. The morphology, surface roughness, and layer thickness of monolayer MoS_2_ before and after baking were thoroughly investigated using atomic force microscopy. The results showed that the surface roughness and layer thickness of monolayer MoS_2_ modified by baking were obviously increased in comparison with MoS_2_ without baking, indicating that the surface topography of the monolayer MoS_2_ film was obviously influenced. Moreover, a photoluminescence spectrum study revealed that p-type doping of monolayer MoS_2_ displayed much greater photoluminescence ability, which was taken as evidence of higher photothermal conversion efficiency. This study not only developed a novel MoS_2_ with high EEE for future biomedical applications but also demonstrated that a baking process is a promising way to modify the surface of monolayer MoS_2_.

## Introduction

Two-dimensional materials (2DMs) have attracted extremely wide attention in the biomedical science field because of their various unique properties (Yin et al., 2016; Liu and Liu, 2018). 2DMs are a large family of materials that include semimetals (graphene), semiconductors (molybdenum disulfide (MoS_2_), black phosphorus, etc.), insulators (h-BN), superconductors (carbon nanotubes), thermoelectric materials (PbTe), and topological insulators (HgTe quantum wells) (Frindt, 1966; Wilson and Yoffe, 1969; Clement et al., 1978; Abruna and Bard, 1982; Mishra et al., 1997; Prasad and Zabinski, 1997; Poizot et al., 2000; Frey et al., 2003; Kane and Mele, 2005). In recent years, another class of 2DMs, transition metal dichalcogenides (TMDCs), have emerged and also received much attention as next-generation applications in electronics and optoelectronics because of their various unique optical, electrochemical, catalytic, and lubrication properties (Radisavljevic et al., 2011a,b; Choi et al., 2012; Wang et al., 2012; Sundaram et al., 2013). The chemical formula of two-dimensional (2D) TMDCs is MX_2_, where M stands for a transition metal element, such as Mo, W, etc., and X stands for a chalcogen element, such as S, Se, etc. (Wu et al., 2016). TMDCs are a series of compounds with layered structures, and the bulk MX_2_ is composed of multiple X–M–X layers that are held in stacks by weak van der Waals forces. The single-layer MX_2_ has a unique sandwich-like structure, with a plane of M atoms wedged into two planes of X atoms. The M and X atoms are held together by strong covalent bonds (Lee et al., 2012). MoS_2_ is a typical 2D TMDC compound, with the height of each layer being 0.65 nm (Eda et al., 2011). In its bulk structure, MoS_2_ is a semiconductor with an indirect bandgap of about 1 eV, while monolayer MoS_2_ has direct bandgap of 1.8 eV (Li and Zhu, 2015). Because of its outstanding properties, MoS_2_ has been widely studied and used in various applications, including transistors, sensors, and memory and optoelectronic devices in the biomedical sciences (Kim et al., 2012; Singh et al., 2016). Recently, MoS_2_-based nanoplatforms have been reported as photothermal agents used for cancer therapy and treatment of bacteria-induced infections because of their good biocompatibility and high photothermal conversion efficiency in the near-infrared region (Chou et al., 2013; Yin et al., 2014, 2016; Gao et al., 2018). Chou et al. (2013) synthesized and prepared MoS_2_ nanosheets that were used in photothermal therapy (PTT) for cancer. The study showed that MoS_2_ nanosheets had much higher photothermal conversion efficiency than graphene and gold nanorods, thereby improving the therapeutic efficacy in cancer therapy (Chou et al., 2013). Inspired by the effective photothermal conversion of MoS_2_, Yin et al. (2016) developed polyethylene glycol-functionalized MoS_2_-based nanoflowers that showed high antimicrobial activity for Gram-negative ampicillin-resistant *Escherichia coli* and Gram-positive endospore-forming *Bacillus subtilis*.

Broadly speaking, the methods to obtain MoS_2_ films include top-down and bottom-up methods, such as micro-mechanical stripping, lithium-ion intercalation, liquid-phase ultrasonic stripping, and chemical vapor deposition (CVD) (Ramakrishna Matte et al., 2010; Coleman et al., 2011; Lee et al., 2012; Baugher et al., 2013; Zhang et al., 2014). The technology of the micro-mechanical stripping method is completely mature; nevertheless, achieving large-scale production using this method remains a challenge (Baugher et al., 2013). The lithium-ion intercalation method is quite time-consuming and has extremely strict requirements for the preparation conditions (Ramakrishna Matte et al., 2010), while the degree and efficiency of the liquid-state ultrasonic stripping method are relatively low and the resulting single-layer product content is low (Coleman et al., 2011). Therefore, it is necessary to optimize large-area deposition methods for MoS_2_ films to scale up these technologies. In recent years, a method of growing MoS_2_ by CVD on insulating substrates has been developed. The CVD method is easy to operate and can achieve high-quality, large-area continuous synthesis batches with high efficiency (Zhang et al., 2014); this method is better suited to industrialization and can quickly adapt to the large-scale application of MoS_2_. In 2012, Lee and co-workers reported the synthesis of large-area monolayers of MoS_2_ thin films on silicon dioxide substrates by CVD for the first time (Lee et al., 2012). Indeed, a native n-doping behavior of not intentionally doped MoS_2_ was confirmed by previous investigations (Mak et al., 2013; Fang et al., 2014) because of hypothetical sulfur vacancies (Qiu et al., 2013; Tongay et al., 2013). The n-type doping limits carrier conduction in MoS_2_ to its conduction bands, and p-type doping is more desirable for MoS_2_-based field-effect transistor devices (Zhang et al., 2013). Inspired by this demand, research on the doping of MoS_2_ has been extensively encouraged (Chuang et al., 2016). The p-type doping of MoS_2_ has lower resistance and better performance than the original MoS_2_ (Laskar et al., 2014; Neal et al., 2017), the basic mechanism of which is to suppress n-type conductivity. Moreover, 2DMs also enable new methods such as surface charge transfer (Zhang et al., 2016) and intercalation (Jung et al., 2016) to be used. But these methods are neither easy nor convenient. Another commonly used doping method is chemical doping. Rhenium and chlorine are used as substitution donors, while niobium and phosphorus are used as substitution acceptors (Tiong et al., 1999; Laskar et al., 2014; Suh et al., 2014; Yang et al., 2014; Das et al., 2015; Park et al., 2015). The p-type doping of MoS_2_ films is carried out by fluorine plasma treatment or the spin-on AuCl_3_ method (Chen et al., 2013; Liu et al., 2016; Xue et al., 2016). In addition, oxygen is frequently used for doping as pure physical adsorption of oxygen will have only a small effect, which can cause an increase in the threshold voltage and a decrease in the on-current; however, the interaction between oxygen and MoS_2_ films is too weak to overcome the intrinsic n-type doping. In this case, some of the intrinsic properties of MoS_2_ can be altered by the oxygen plasma method (Nan et al., 2014), which is widely used to prepare p-type doping of MoS_2_. However, the aforementioned methods of preparing p-type doping of MoS_2_ have some limitations. (i) There is residual organic solvent after chemical doping. (ii) Plasma treatment is carried out under harsh reaction conditions and requires careful control of the power to decrease damage to the sample surface. (iii) In addition, spin-on AuCl_3_ is mainly used for achieving high performance in p-type field-effect transistors (Park et al., 2015; Xue et al., 2016).

Therefore, it is of great importance to efficiently and effectively synthesize and prepare p-type doping of MoS_2_ films to enhance the energy exchange efficiency (EEE) for PTT in biomedical applications. In this work, we report a simple and convenient method for the preparation of p-type doping of monolayer MoS_2_ through baking under ambient conditions. The monolayer MoS_2_ film was synthesized using the CVD method first. Optical microscopy (OM) and atomic force microscopy (AFM) were used to directly image and evaluate the morphology associated with monolayer MoS_2_ grown by CVD onto a SiO_2_/Si substrate. Then, the monolayer MoS_2_ film was modified via baking at different temperatures under ambient conditions. Finally, the physicochemical properties of the modified monolayer MoS_2_ film were thoroughly investigated using AFM, Raman spectroscopy, photoluminescence (PL), and X-ray photoelectron spectroscopy (XPS). This study not only prepared a p-type doping MoS_2_ film with high EEE, but also demonstrated that the baking process is a promising way to modify the surface of an MoS_2_ film, which encourages further investigations for biomedical applications.

## Materials and Methods

### Materials

N-type silicon covered with 30 nm silicon dioxide was purchased from Suzhou Ruicai Semiconductor Co. Ltd. MoO_3_ (AR) was obtained from Nanjing Muke Nanotechnology Co. Ltd. S powder (99.95 %) was ordered from Aladdin Reagent Co. Ltd. Ar (99.99%) was purchased from Harbin Dawn Gas. Acetone, anhydrous ethanol, and deionized water were obtained from Wuxi Ledong Microelectronics Co. Ltd. The CVD oven was purchased from Hefei Kejing Material Technology Co. Ltd.

### Synthesis of MoS_2_ Films

MoS_2_ films were synthesized using the CVD method. N-type silicon covered with 30 nm silicon dioxide was used as the substrate to grow the MoS_2_ films. A silicon wafer was cleaned through sonication for 10 min with acetone, anhydrous ethanol, and deionized water, respectively. Subsequently, after drying under argon, the silicon wafer was further cleaned by plasma oxygen cleaner. MoO_3_ and elemental S powder were used as the precursor and reactant materials, respectively, to grow a single layer of MoS_2_ at a temperature of 700°C under atmospheric pressure. The whole experiment was carried out under Ar. MoS_2_ films were obtained after 25 min.

### Baking of the MoS_2_ Film

Four monolayer MoS_2_ samples was prepared under the same conditions using the CVD method. One was used as a control sample without any further treatment. The other three were baked under atmosphere in an oven for 2 h, at 150, 200, and 250°C, respectively.

### X-Ray Photoelectron Spectroscopy Analysis

The control sample and the MoS_2_ sample baked at 250°C were prepared for XPS. Using X-rays to radiate the samples, spectra can be obtained after software processing. XPS was performed to verify whether MoS_2_ can form an Mo–O bond via baking.

### Optical Microscopy Imaging

The number of layers of MoS_2_ films was confirmed by OM. A silicon wafer with MoS_2_ films was placed on a glass slide, which was put on the objective stage with two clamps to hold it. An appropriate objective was used to observe the MoS_2_ films with a clear view. The OM image can be displayed by a CCD camera mounted on the OM.

### Atomic Force Microscopy Imaging

AFM was carried out on MoS_2_ films on a silicon wafer to characterize the morphology, roughness, and thickness of the samples. An AFM tapping mode (Dimension FastScan, Bruker) was used to scan the samples.

### Raman Spectroscopy Analysis

Raman spectroscopy was carried out on the MoS_2_/Si samples at room temperature with a 532 nm laser as the excitation light source and the laser power was limited to 50 μW to prevent self-heating-induced damage during the measurement. Raman spectra were observed using a micro-confocal Raman spectrometer (Horiba).

### Photoluminescence Analysis

In order to determine the luminescence of MoS_2_ before and after baking, PL was carried on the monolayer MoS_2_ at different baking temperatures. PL spectra were observed using the same machine as used for the Raman spectra. The only difference was that during Raman measurement the sample was placed directly in the machine at room temperature under air, while during PL measurement the sample was placed in a cryostat.

## Results and Discussions

### Preparation and Characterization of MoS_2_ Films

**Figure 1:**
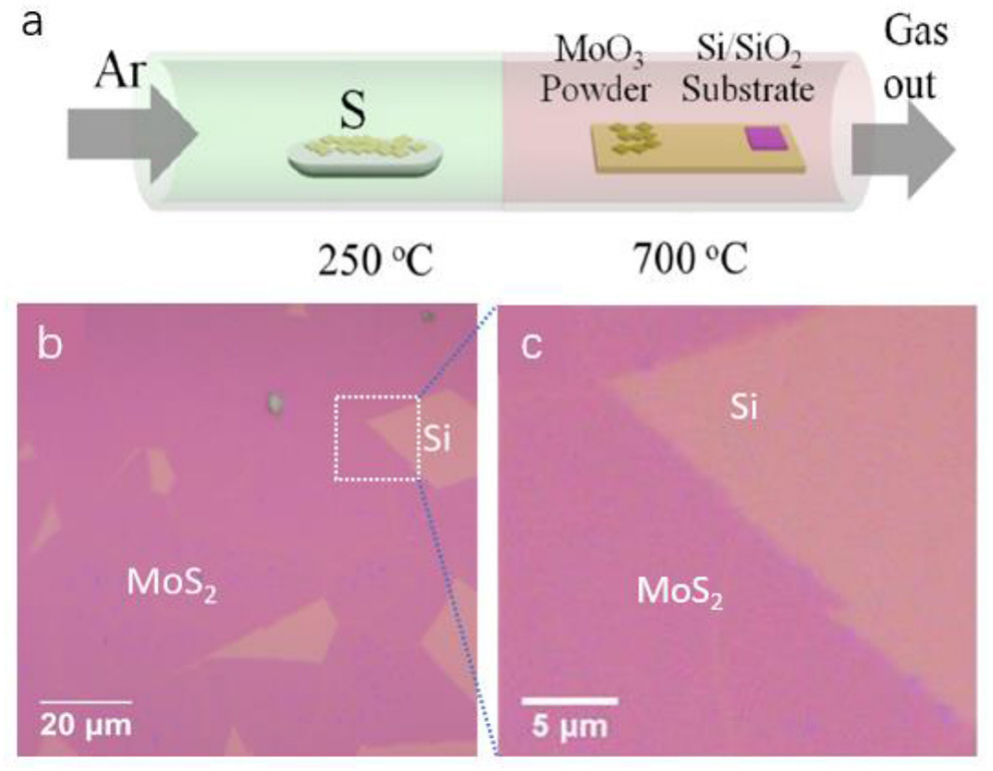
**(A)** A schematic of the synthesis of a monolayer MoS_2_ film using the chemical vapor deposition (CVD) method. **(B,C)** Optical micrographs of monolayer MoS_2_ prepared by the CVD method on a silicon substrate. **(B)** Scale bar, 20 μm. **(C)** Scale bar, 5 μm.

The monolayer MoS_2_ film was prepared by the CVD method, as shown in Figure 1A. The n-type silicon substrate covered with 30 nm silicon dioxide was cleaned by plasma oxygen cleaner, followed by the growth of the monolayer MoS_2_ film using MoO_3_ and elemental S powder as the precursor and reactant materials, respectively. The reaction was carried out at 700°C at atmospheric pressure under Ar for 25 min. Representative OM images of the as-deposited samples are shown in Figures 1B,C, demonstrating the presence of large MoS_2_ domains with uniform and smooth morphology, as indicated by the homogeneous purple color on the silicon substrate (Castellanos-Gomez et al., 2010; Li et al., 2012). Some irregular parts of the silicon wafer (orange) were exposed that were not covered with MoS_2_ film because of the rupture of the monolayer MoS_2_ film during the CVD process. Collectively, the monolayer MoS_2_ film was successfully prepared by the CVD method on the silicon substrate.

### Study of the Baking Effect on the MoS_2_ Film

#### Atomic Force Microscopy Imaging

**Figure 2:**
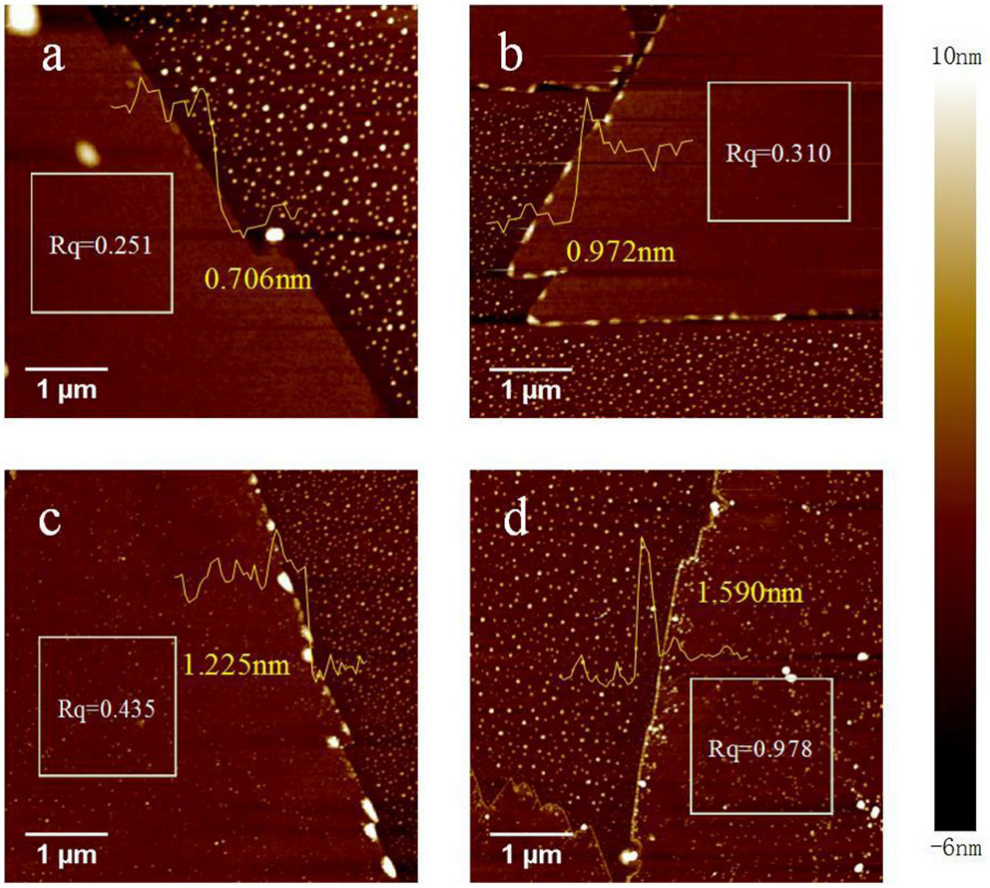
Atomic force microscopy images of the monolayer MoS_2_ film before and after baking at different temperatures for 2 h under air. **(A)** The original monolayer MoS_2_ film prepared by the chemical vapor deposition method. The monolayer MoS_2_ film baked at **(B)** 150°C, **(C)** 200°C, and **(D)** 250°C. The yellow line indicates the surface height of the monolayer MoS_2_ film. The area indicated with the white square was measured to confirm the roughness. Scale bar, 1 μm.

Next, we treated the monolayer MoS_2_ film via baking at 150, 200, or 250°C for 2 h under air, followed by imaging through AFM. To further confirm the morphology and size (especially the thickness) of the monolayer MoS_2_ film, the border of the MoS_2_ film and wafer was imaged, as shown in Figure 2. The areas with white dots are the silicon wafer, and the integrated and smooth areas are the monolayer MoS_2_ film. To further characterize the morphology and size of the samples, the perpendicular height (thickness) of the monolayer MoS_2_ film and roughness of the area indicated by the white square were measured. Figure 2A shows a higher resolution tapping mode AFM morphological image of an MoS_2_ domain partially covering the SiO_2_ surface without the baking process. A uniform and smooth MoS_2_ film was observed, and the result was consistent with that of OM imaging. The thickness and roughness (*R*_q_) were, respectively, confirmed as 0.706 nm and 0.251, which further proved the successful synthesis of the monolayer MoS_2_ film (Giannazzo et al., 2020). After baking at 150°C (Figure 2B), the thickness and the *R*_q_ of the treated MoS_2_ film were increased to 0.972 nm and 0.310, respectively. Moreover, after baking at 200°C (Figure 2C) and 250°C (Figure 2D), the thickness was obviously increased to 1.225 nm and 1.590 nm, respectively. In addition, the *R*_q_ was significantly increased (0.435 at 200°C and 0.978 at 250°C). These results show that the thickness and roughness of the monolayer MoS_2_ film were increased with an increase in baking temperature. In addition, the surface topography of the sample was also changed with an increase in the baking temperature. Compared with the original sample, the monolayer MoS_2_ film baked at 150°C still showed a smooth and clear surface, indicating no obvious changes in the surface topography. However, some white dots were observed in the sample after baking at 200°C, and denser and larger white dots appeared on the surface of the sample after baking at 250°C. These changes possibly resulted in the increase in the roughness of the monolayer MoS_2_ film. As reported previously, the white dots could be the etched parts of the monolayer MoS_2_ flake, which would break down at 300°C (Zhou et al., 2013). This can be explained by the composition of the surface of the MoS_2_ film being disturbed or changed, possibly as a result of a chemical reaction that led to the adsorption or formation of elements. In this etching process, some sulfur vacancies might arise, followed by adsorption and reaction with the oxygen in the air and water vapor, finally resulting in p-type doping of the monolayer MoS_2_ film. In summary, the thickness and surface topography of the monolayer MoS_2_ film were obviously influenced by the baking process and were dependent on the baking temperature.

#### Raman Spectroscopy Analysis

**Figure 3:**
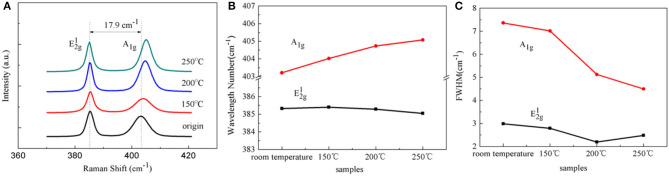
**(A)** Raman spectra of the samples before and after baking at different temperatures for 2 h under air. The wavelength number **(B)** and full width at half-maximum **(C)** of the *A*_1g_ and $E_{2\text{g}}^{1}$ modes before and after baking at 150, 200, and 250°C for 2 h under air.

To further investigate the effect of baking on the monolayer MoS_2_ film, the Raman spectra of samples before and after baking at different temperatures were measured, as shown in Figure 3. Two characteristic vibrational peaks were observed for the MoS_2_ film, namely the in-plane vibration mode $E_{2\text{g}}^{1}$ (385 cm^−1^) and the out-of-plane vibration mode *A*_1g_ (403 cm^−1^) (Figure 3A). The wavenumber difference (Δ = 17.9 cm^−1^) between the peaks' positions is consistent with the presence of the monolayer MoS_2_ domains (Kim et al., 2018). Figures 3B,C shows the changes in the frequency shift and full width at half-maximum (FWHM) of two modes with the increase in the baking temperature, respectively. With the increase in the baking temperature, no obvious shift was observed for the $E_{2\text{g}}^{1}$ mode, whereas the *A*_1g_ mode showed a right shift with an increase of ~2 cm^−1^, from about 403 cm^−1^ to 405 cm^−1^ (Figures 3A,B). The different changes in the $E_{2\text{g}}^{1}$ and *A*_1g_ modes demonstrated the presence of p-type doping of MoS_2_ (Chakraborty et al., 2012; Mao et al., 2013). Moreover, the FWHM of the *A*_1g_ mode was obviously decreased with the increase in the baking temperature. However, the $E_{2\text{g}}^{1}$ mode showed negligible changes compared with those of the *A*_1g_ mode with the increase in the baking temperature. As reported previously, both the doping and strain showed an effect on the vibrational modes of the Raman spectra of the monolayer MoS_2_ film (Scalise et al., 2014). If the changes were induced by strain, Raman modes $E_{2\text{g}}^{1}$ and *A*_1g_ would shift together, compressive strain could cause blue shift of $E_{2\text{g}}^{1}$ and *A*_1g_, tensile strain could cause red shift of $E_{2\text{g}}^{1}$ and *A*_1g_, therefore there was no strain effect in above results. In contrast, if the changes were caused by doping, the wavenumber and the FWHM of the *A*_1g_ mode were obviously changed, and the $E_{2\text{g}}^{1}$ mode was maintained stable. Furthermore, blue shift of the *A*_1g_ mode and a decrease in the FWHM corresponded to p-type doping, and red shift and an increase in the FWHM corresponded to n-type doping (Chakraborty et al., 2012). In this case, the results revealed that the p-type doping of the MoS_2_ film was caused by the baking process.

#### X-Ray Photoelectron Spectroscopy Analysis

**Figure 4:**
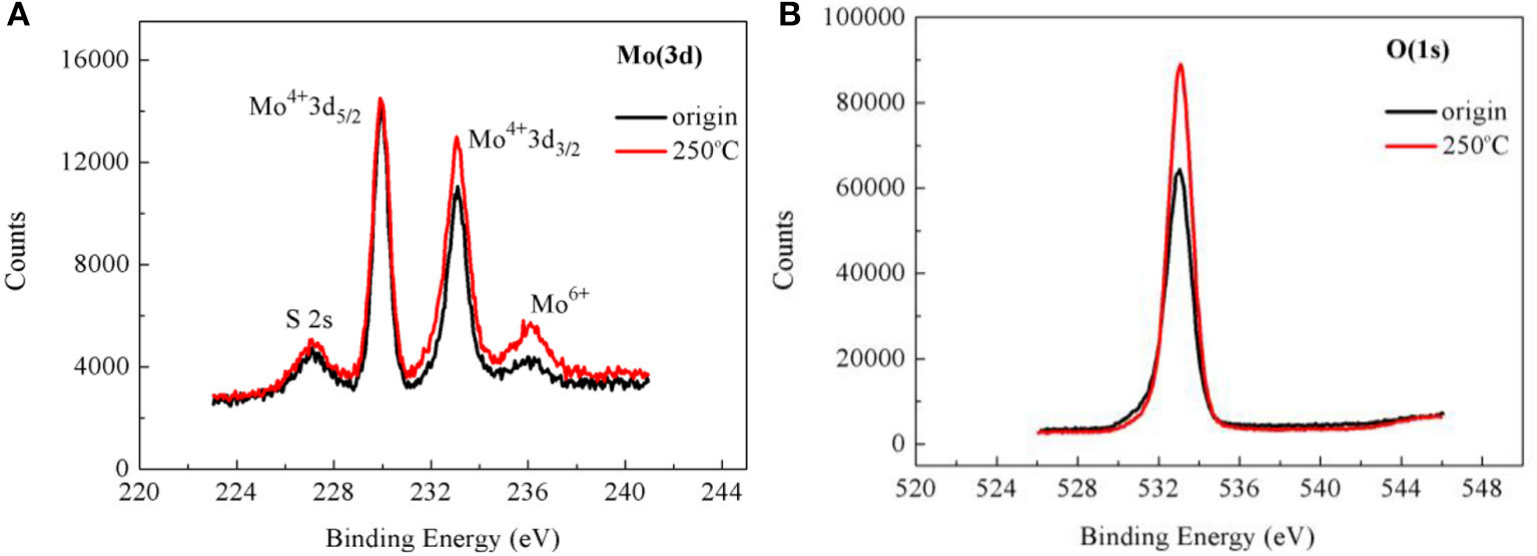
X-ray photoelectron spectroscopy (XPS) spectra of the monolayer MoS_2_ film before and after baking at 250°C for 2 h under air. **(A)** The XPS spectra of Mo(3d) atoms in the samples before and after baking. **(B)** The XPS spectra of O(1s) atoms in the samples before and after baking.

To further confirm the p-type doping of MoS_2_ after baking, XPS spectra of samples in the Mo(3d) and O(1s) regions before and after baking at 250°C for 2 h under air were monitored, as shown in Figures 4A,B, respectively. First, the peaks at S 2s, Mo^4+^ 3d_5/2_, and Mo^4+^ 3d_3/2_ were observed (Figure 4A), showing the successful synthesis of the monolayer MoS_2_ film. After the baking process, the signals of the Mo^4+^ 3d_3/2_ and Mo^6+^ peaks were significantly enhanced (Figure 4A), indicating the generation of MoO_3_, which proved the p-type doping of MoS_2_. Furthermore, the signal of the O(1s) peak of the sample after baking was also significantly enhanced in comparison with that of the original sample (Figure 4B), implying p-type doping of MoS_2_ after the baking process. This can be explained by elemental oxygen being adsorbed on the surface of the sample, followed by reaction with the S vacancy during the baking process. In summary, the results of the XPS spectra further confirmed the presence of p-type doping of MoS_2_ after the baking process.

#### Photoluminescence Analysis

**Figure 5:**
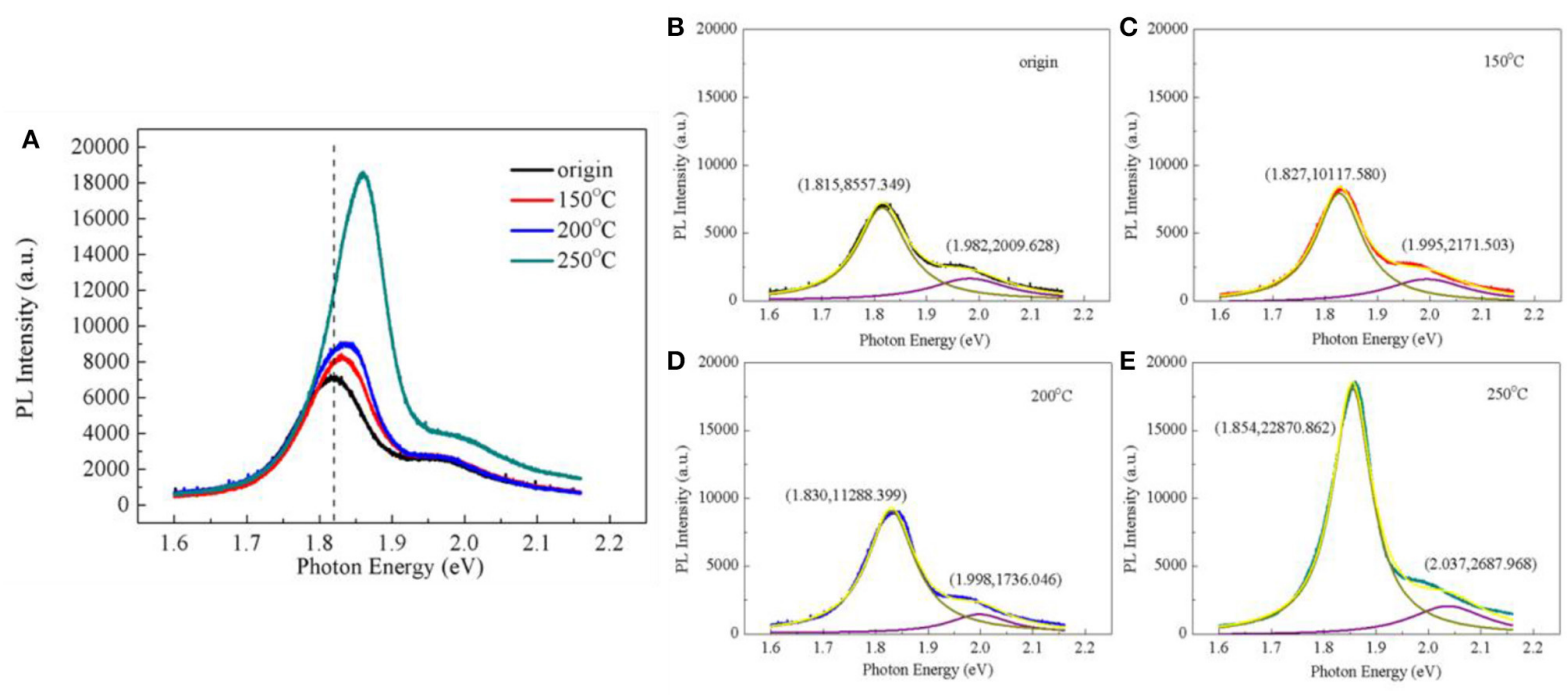
**(A)** Photoluminescence (PL) spectra of the monolayer MoS_2_ film before and after baking at different temperatures. PL peak fitting spectra of the original sample **(B)** and after baking at 150°C **(C)**, 200°C **(D)**, and 250°C **(E)**.

Since we successfully synthesized the monolayer MoS_2_ film and prepared the p-type doping of the MoS_2_ film, we next evaluated whether the luminescence of MoS_2_ was enhanced after the baking process using PL measurement (Figure 5). Figure 5A shows the PL spectrum of the monolayer MoS_2_ film before and after baking at different temperatures. There are two major peaks in the PL spectrum, which correspond to A1 and B1 excitons, respectively. The two peaks are associated with the direct excitonic transitions at the Brillouin zone K point. The energy difference between the two peaks is due to the spin-orbital splitting of the valence band. These PL characteristics are consistent with previous work (Coehoorn et al., 1987a,b; Mak et al., 2010; Splendiani et al., 2010). Therefore, the PL spectra of the samples were divided into two peaks, where (X, Y) represents the nature of the curve after each subpeak. X represents the center of the peak, and Y represents the overall height of the peak as well as the luminous intensity of the sample, as shown in Figures 5B–E. For the first peak in the PL spectra, the PL intensity was enhanced step by step with the increase in the baking temperature. In particular, the PL intensity was obviously enhanced after baking at 250°C, demonstrating a 2.7-fold higher intensity than that of the original sample. The shape of the PL spectrum also changed after baking, becoming sharper with the baking treatment. The first peak energy was right (blue) shifted with an increase in the baking temperature. The right (blue) shift and the enhancement of the PL intensity could be explained by the contributions of the exciton and the charged exciton (trion) in the first peak. As previous works have shown, the charged exciton transformed to an exciton after the baking process, and the exciton was dominant in the first peak, which induced the changes in the PL spectrum. The combination of the two peaks corresponding to the A1 and B1 excitons improved the PL capacity compared with the original sample (Mak et al., 2013; Mouri et al., 2013). In addition, the unique sandwich-like structure (S–Mo–S triatomic layer) also increased the PL ability of MoS_2_ after baking (Splendiani et al., 2010; Coleman et al., 2014). As mentioned above, during the baking process, the surface of the MoS_2_ was active for air adsorption, which was manifested in the active physical adsorption of oxygen and water vapor. In addition, Mo–O bonds and MoO_3_ might be generated by the chemical reactions, facilitating the transformation of the charged exciton to the exciton, thereby leading to the enhanced PL intensity and higher EEE (Wei et al., 2014). In conclusion, these results proved that the p-type doping of MoS_2_ prepared by the baking process exhibited much higher EEE than the control sample, in which p-type doping of MoS_2_ might be a promising nanoplatform for PTT in biomedical science.

## Conclusions

In this work, we successfully prepared a monolayer MoS_2_ film using the CVD method. Following this, OM and AFM were used to characterize the morphology of the sample. Furthermore, a baking process was utilized to treat the monolayer MoS_2_ film at different temperatures under air. The surface topography of the monolayer MoS_2_ film before and after the baking process was investigated by AFM, especially the thickness and roughness. Subsequently, the Raman and XPS spectra of the samples were used to confirm p-type doping of the monolayer MoS_2_ film and the mechanism of generation through baking. To further evaluate the energy exchange capacity, the PL of the samples before and after the baking process was measured, and the results showed that p-type doping of the monolayer MoS_2_ film exhibited much higher EEE than the original control. This study not only developed a monolayer MoS_2_ film with high EEE that might be a promising platform for PTT or imaging in biomedical applications, but also showed that the baking process could be a convenient method to prepare p-type doping of MoS_2_ with improved properties by surface modification.

## Data Availability Statement

The raw data supporting the conclusions of this article will be made available by the authors. Additional data related to this paper may be requested from the authors.

## Author Contributions

YW and YM: methodology and investigation. XY: software. JL and HZ: validation. YW and JL: formal analysis. YW: resources. JS: data curation. YW and JS: writing-original draft preparation. CZ: writing-review and editing. YW and XY: visualization. JL, KJ, and CZ: supervision. JL: project administration and funding acquisition. All authors contributed to the article and approved the submitted version.

## Conflict of Interest

The authors declare that the research was conducted in the absence of any commercial or financial relationships that could be construed as a potential conflict of interest.
